# Interleukin-6 Induces Gr-1+CD11b+ Myeloid Cells to Suppress CD8+ T Cell-Mediated Liver Injury in Mice

**DOI:** 10.1371/journal.pone.0017631

**Published:** 2011-03-04

**Authors:** Liang Cheng, Jun Wang, Xiaozhu Li, Qiao Xing, Peishuang Du, Lishan Su, Shengdian Wang

**Affiliations:** 1 Key Laboratory of Infection and Immunity, Institute of Biophysics, Chinese Academy of Sciences, Beijing, China; 2 Graduate University, Chinese Academy of Sciences, Beijing, China; 3 Department of Microbiology and Immunology, School of Medicine, Lineberger Comprehensive Cancer Center, University of North Carolina at Chapel Hill, Chapel Hill, North Carolina, United States of America; University of Colorado Denver, United States of America

## Abstract

**Background:**

Agonist antibodies against CD137 (4–1BB) on T lymphocytes are used to increase host anti-tumor immunity, but often leading to severe liver injury in treated mice or in patients during clinical trials. Interleukin-6 (IL-6) has been reported to protect hepatocyte death, but the role of IL-6 in protecting chronic T cell-induced liver diseases is not clearly defined due to lack of relevant animal models. We aimed to define the role of IL-6 in CD8+ T cell-mediated liver injury induced by a CD137 agonistic mAb (clone 2A) in mice.

**Methods/Principal Findings:**

We expressed IL-6 in the liver by hydrodynamic gene delivery in mice treated with 2A or control mAb and studied how IL-6 treatment affected host immunity and T cell-mediated liver injury. We found that ectopic IL-6 expression in the liver elevated intrahepatic leukocyte infiltration but prevented CD8+ T cell-mediated liver injury. In IL-6 treated mice, CD8+ T cells proliferation and IFN-γ expression were inhibited in the liver. We discovered that IL-6 increased accumulation of Gr-1+CD11b+ myeloid derived suppressor cells (MDSCs) in the liver and spleen. These MDSCs had the ability to inhibit T cells proliferation and activation. Finally, we showed that the MDSCs were sufficient and essential for IL-6-mediated protection of anti-CD137 mAb-induced liver injury.

**Conclusions/Significance:**

We concluded that IL-6 induced Gr-1+CD11b+ MDSCs in the liver to inhibit T cell-mediated liver injury. The findings have defined a novel mechanism of IL-6 in protecting liver from CD8+ T cell-mediated injury.

## Introduction

Hepatitis or liver inflammation is a common disease, mainly caused by hepatitis B or C viruses, alcohol, and various chemical agents. In addition to direct hepatocyte killing, both innate and adaptive immune cells contribute to various forms of liver injury, and CD8+ T cells or cytotoxic T lymphocytes (CTLs) are likely the main effectors for virus-induced hepatitis[1].

CD137 (4-1BB) is an inducible co-signaling receptor belongs to the TNF receptor superfamily, which is found on activated T cells, NK cells, dendritic cells, and macrophages[2]. Engagement of CD137 provides a costimulatory signal to induce T-cell expansion, IFN-γ production, and prevention of activation-induced death of effector T cells[3]. In the absence of TCR triggering, CD137 stimulation induces vigorous growth of both CD8+ and CD4+ T cells with memory phenotype [4]. Treatment with an agonistic anti-CD137 antibody (clone 2A) in mice can cause CD8+ T cell-dependent tumor rejection and virus clearance [5], [6]. Recently, using the agonist 2A mAb as a mimicry of CD137L, we set up a new model for CD8+ T cell-mediated liver injury[7]. A single 2A treatment triggers hepatic infiltration and activation of CD8+ T cells, and CD8+ T cell-derived IFN-γ plays a central role in the liver injury[7]. Clinical studies also show that anti-CD137 antibody treatment in some cancer patients leads to liver toxicity, which leads to suspension of current clinical trials[8], [9].

As a pleiotropic cytokine, IL-6 is implicated in both proinflammatory and anti-inflammatory responses[10]. In models of chronic inflammatory diseases, such as arthritis, colitis, or experimental autoimmune encephalomyelitis, IL-6 plays a proinflammatory role [11]–[14]. Through continuous MCP-1 induction, it accelerates mononuclear cell accumulation at the site of inflammation. And it also can promote angioproliferation and antiapoptotic functions on T cells [10], [15]. While in models of acute inflammation, it exhibits an anti-inflammatory profile[16]. IL-6 also has complicated role in the liver. It is recognized as a hepatocyte-stimulating factor [17]. During acute and chronic liver disease, the expression of IL-6 correlates with liver disease progression [18]. IL-6 could promote liver regeneration [19]–[21] and protects hepatocyte death induced by concanavalin A (ConA), anti-Fas, alcohol, acetaminophen, or carbon tetrachloride mediated hepatic damage through the signal transducer and activator of transcription 3 (STAT3) pathway after engaging the heterodimeric gp80/gp130 receptor [22]–[29]. It is believed that this hepatic protective effect is mediated either by the induction of anti-apoptotic proteins including Bcl-xL, Bcl-2, and FLIP in the hepatocytes, or by inducing of soluble factors that regulating immune function such as serum amyloid A2 and keratinocyte growth factor[28], [29]. In addition, IL-6 may also have direct effects on immune cells during hepatic injury. A recent study shows that, in ConA-mediated hepatitis, IL-6 can also inhibit the activity of NKT cells in a CD4+ T cell and STAT3-dependent manner [30]. However, due to the lack of a model for CD8+ T cell-mediated chronic hepatitis, whether and how IL-6 plays a role in CTL mediated hepatitis is still unclear.

Gr-1+CD11b+ myeloid derived suppressor cells (MDSCs) are a heterogeneous cell population consists of immature myeloid cells and myeloid progenitor cells that could suppress immune responses by a variety of mechanisms[31]. Interestingly, IL-6 receptor is expressed on Gr-1+CD11b+ myeloid cells [32]. In a mouse tumor model, the IL-1R-deficient mouse has a delayed accumulation of MDSCs, which can be partially restored by IL-6[32]. Thus IL-6 may induce Gr-1+CD11b+ myeloid cells expansion in some pathological circumstance.

It is of interest that IL-6 is significantly elevated in patients with acute and chronic liver diseases [18], but its role in CTL-mediated hepatitis has not been defined. In this study, we investigated the role of IL-6 in CD8+ T cell-mediated hepatitis triggered by anti-CD137 mAb. We showed that IL-6 could prevent CD8+ T cell-mediated liver injury. Mechanistically, IL-6 induced accumulation of Gr-1+CD11b+ myeloid cells in the liver, which suppressed T cell activation to prevent liver injury.

## Results

### Intrahepatic IL-6 expression in vivo leads to enhanced liver leukocytes infiltration and splenomegaly but prevents T cell-mediated liver injury

**Figure 1:**
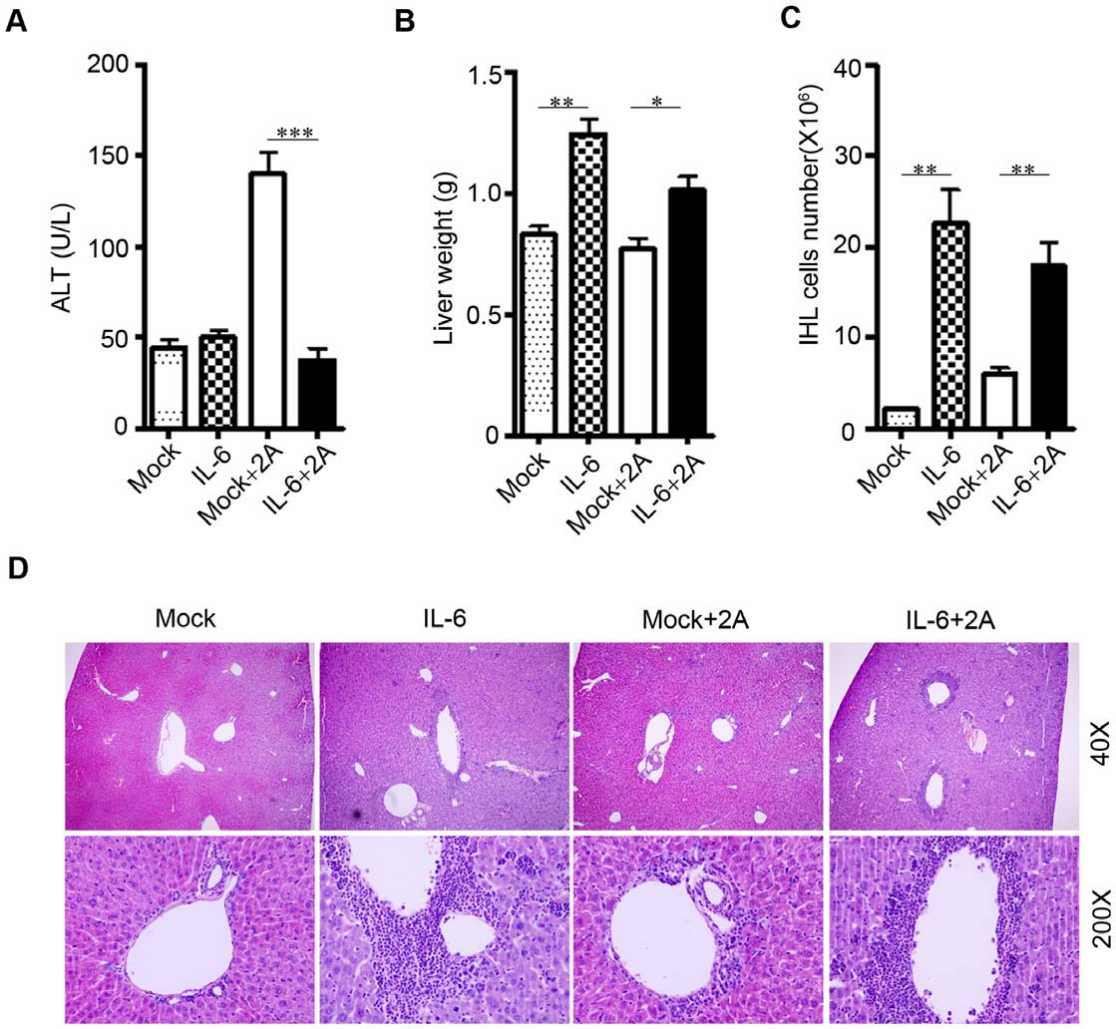
Ectopic IL-6 expression in vivo leads to enhanced intrahepatic leukocytes infiltration but protect T cell-mediated liver injury. C57BL/6 mice were hydrodynamic injected with pcDNA-IL6 plasmid or mock control plasmid at day 0. At day 2, mice were further injected i.p. with 100 µg 2A antibody or or control antibody (RIg).The mice were sacrificed at day 12, and serum ALT levels (A), liver weight (B) and the number of intrahepatic leukocytes (C) were measured. (D) At day12, the livers were fixed and stained with H&E. Representative images from 3–4 mice in each group were shown. Three experiments were performed with similar results. *P<0.05, **P<0.01, ***P<0.001 in comparison with groups as indicated.

We have recently established a CD8+ T cell-mediated hepatitis model induced by a CD137 agonistic mAb (clone 2A) [7]. To evaluate the potential effect of IL-6 in CD8+ T cell-mediated chronic hepatitis, we tested the role of IL-6 in the liver inflammation model induced by 2A[7]. As reported [7], a single treatment of 2A led to significant infiltration of leukocytes into the liver in mice **(Figure S1A)**. The CD8+ T cells were preferentially increased in the liver 10 days after 2A treatment **(Figure S1B–C)**. We expressed IL-6 protein in mice by hydrodynamic injection. Two days after injection of IL-6 or vector control plasmid, the mice were injected with 100 µg of 2A or control antibodies and mice were sacrificed at day 12.After treatment with 2A, we observed liver injury as indicated by the elevated serum level of alanine aminotransferase (ALT) in mice **(** **Figure 1A** **)**. Interestingly, the 2A treated mice showed no elevated blood ALT or liver injury if mice were also treated with IL-6 **(** **Figure 1A** **).** Increase of intrahepatic leukocytes (IHLs) often correlates with liver inflammation and injury. 2A treatment increased IHLs by about 2-fold **(** **Figure 1C** **)**. However, we found enlarged livers in the IL-6 treated mice with a 10-fold increase in IHL infiltration but no obvious liver injury **(** **Figure 1A–D** **)**. In vivo expression of IL-6 also led to obvious splenomegaly **(Figure S2A–B)**, consistent with the clinical observation that IL-6 serum concentration correlated with the spleen size as determined by ultrasound in patient with chronic liver disease [18]. These results raise intriguing question as to how massive increase of IHLs by IL-6 led to no sign of liver injury, even suppressed 2A mediated liver injury.

### Proliferation and activation of CD8+ T cells are inhibited in IL-6 treated mice

**Figure 2:**
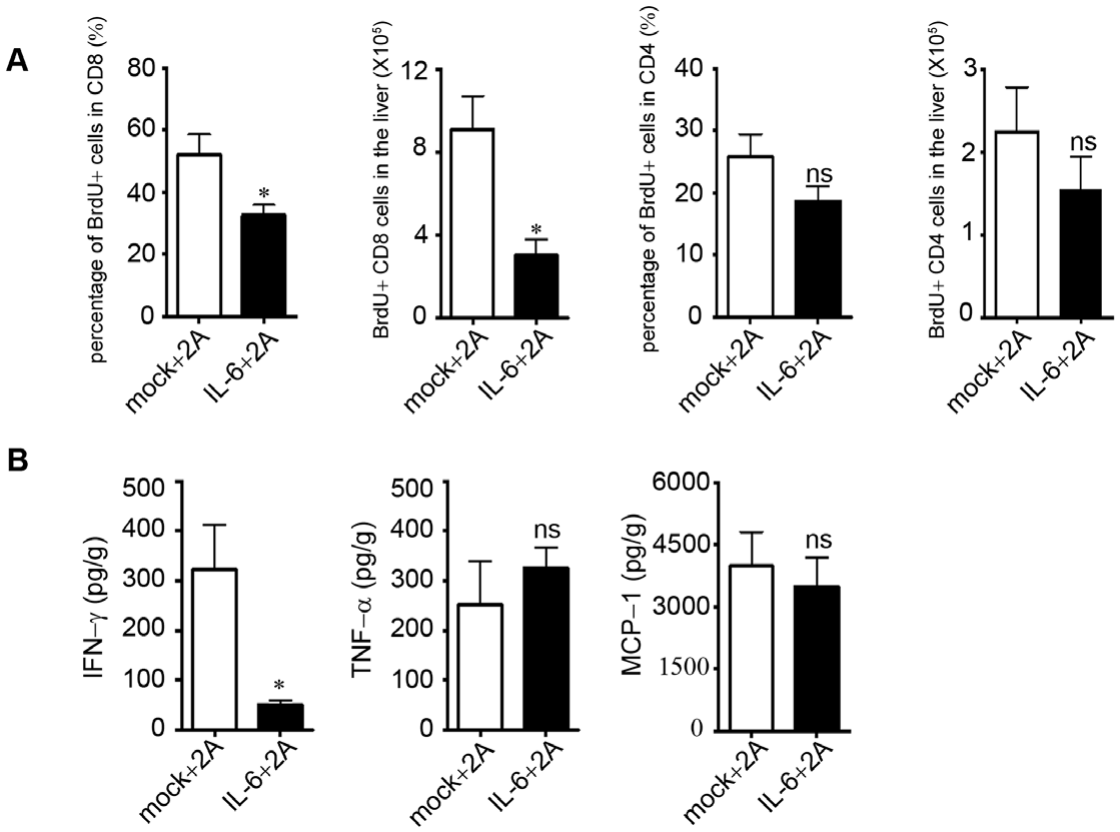
Proliferation and activation of CD8+ T cells were inhibited in vivo in IL-6 treated mice. C57BL/6 mice were treated with IL-6 plasmid and 2A as described in Figure 1 and further fed with drinking water containing 0.8 mg/mL of BrdU from day7 to day12. (A)At day12, the intrahepatic leukocytes were harvested and BrdU incorporation was analyzed by FACS. Percentage of BrdU+ CD8+ T cells in total CD8+ T cell and total number of BrdU+ CD8+ T cells in liver were shown. (B) The cytokine levels in the liver homogenate were compared. Representative data from 2 independent experiments with at least 3 mice per group is shown. *P<0.05 in comparison with control groups.

CD8+ T cell-derived IFN-γ plays a central role in 2A-induced liver injury [7], [33]. We investigated whether IL-6 treatment inhibited the activation of CD8+ T cell in vivo. After IL-6 and 2A treatment, mice were fed with BrdU for 5 days. The IHLs and splenocytes were harvested and analyzed for BrdU incorporation at day 10. We found the IL-6 treated mice showed significant reduction of BrdU+ CD8+ T cells in both liver **(** **Figure 2A** **)** and spleen **(Figure S3)**. BrdU+ CD3+CD8− T cells (CD4+T cells) in the liver showed no significant difference between the two groups **(** **Figure 2A** **)**. In addition, IFN-γ levels in the liver were also remarkably decreased by about 6 fold **(** **Figure 2B** **)**. When we measured the level of TNF and MCP-1 in the liver, however, we found no significant difference between IL-6 and the control group **(** **Figure 2B** **)**. It is possible that IL-6 expression or hydro-dynamic injection may stimulate expression of TNF-α and MCP-1, which was not sufficient to induce liver injury. Therefore, IL-6 expression in the liver suppressed liver injury, correlated with reduced proliferation of CD8+ T cells and IFN-γ expression.

### IL-6 preferentially increases Gr-1+CD11b+ myeloid cells in the liver and spleen

**Figure 3:**
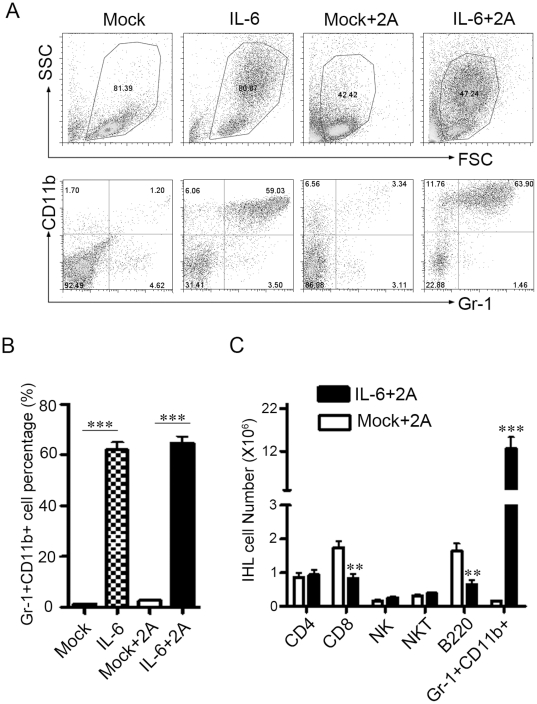
Gr1+CD11b+ myeloid cells are dramatically increased in the liver. C57BL/6 mice were treated as in Figure. 1. At day 12 the intrahepatic leukocytes were isolated for FACS analysis. (A) Representative dot plots of Gr-1+CD11b+ cells in the liver. (B) The percentage of Gr-1+CD11b+ cells in intrahepatic leukocytes in all treated groups. (C) The number of CD4+ T cells, CD8+ T cells, NKT cells, NK cells, B cells, and Gr-1+CD11b+ cells in the liver of 2A treated mice. Three independent experiments were performed with similar results. **P<0.01, ***P<0.001 in comparison with control groups or groups as indicated.

We speculated that IL-6 may induce cells in the liver with immune suppressive functions. We next analyzed the intrahepatic leukocytes by flow cytometry after IL-6 and 2A treatment. As shown in **Figure 3**, about 60% of IHLs were indeed found to be Gr-1+CD11b+ cells in the IL-6 treated mice, while only about 3% in the control treated group. This was consistent with the H&E stained liver sections showing the polymorphonuclear feature in most of the infiltrating leukocytes in the liver of IL-6 treated mice (**Figure 1D** **)**. The number of intrahepatic CD4+ T cells (including FoxP3+CD4+ Treg, data not shown), NK and NKT cells was not significantly different between the IL-6 and control groups. However, we found that CD8+ T and B cell numbers decreased significantly by IL-6 treatment **(** **Figure 3C** **)**. Similar observations were found in the spleen **(Figure S4)**.

### Gr-1+CD11b+ myeloid cells induced by IL-6 inhibit T cells proliferation and activation in vitro

**Figure 4:**
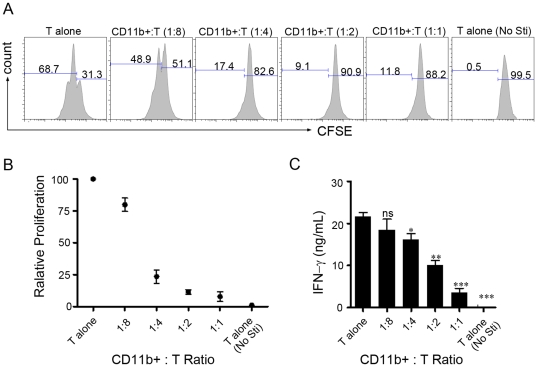
Gr-1+CD11b+ myeloid cells inhibit T cells proliferation and activation in vitro. Purified 3×10^5^CFSE labeled T cells were cultured with 6 µg/mL anti-CD3, in the presence of different ratio of purified CD11b+Gr-1+ cells. CD11b+ cells purification procedure was described in *Materials and Methods*. 60 hour later, the cells were harvested and analyzed by FACS. (A) Representative histograms showing the proliferation of T cells with different ratios of purified CD11b+Gr-1+ cells. (B–C) The relative proliferation and IFN-γ production were measured by CFSE dilution or ELISA, respectively. Proliferation of T cells without CD11b+ cells was set as 100%. The experiment was performed in triplicates. *P<0.05, **P<0.01, ***P<0.001 in comparison with T cells alone.

Given that Gr-1+CD11b+ myeloid cells from tumor-bearing mice could inhibit T cell activation; we investigated if IL-6 induced Gr-1+CD11b+ myeloid cells can inhibit T cell function. Purified Gr-1+CD11b+ cells from IL-6-treated mice were added to CFSE-labeled T cells stimulated with anti-CD3. We found that the Gr-1+CD11b+ cells efficiently inhibited CD3-mediated T cells proliferation **(** **Figure 4A–B** **)**. The Gr-1+CD11b+ cells also suppressed IFN-γ expression in T cells **(** **Figure 4C** **)**. In addition, IL-6 induced Gr-1+CD11b+ cells could also suppressed ConA induced T cell activation in vitro (data not shown). Thus IL-6-induced Gr-1+CD11b+ cells possessed suppressive activity to inhibit T cells activation and proliferation.

### Gr-1+CD11b+ cells induced by IL-6 are sufficient and essential to protect liver from T cell-mediated injury

**Figure 5:**
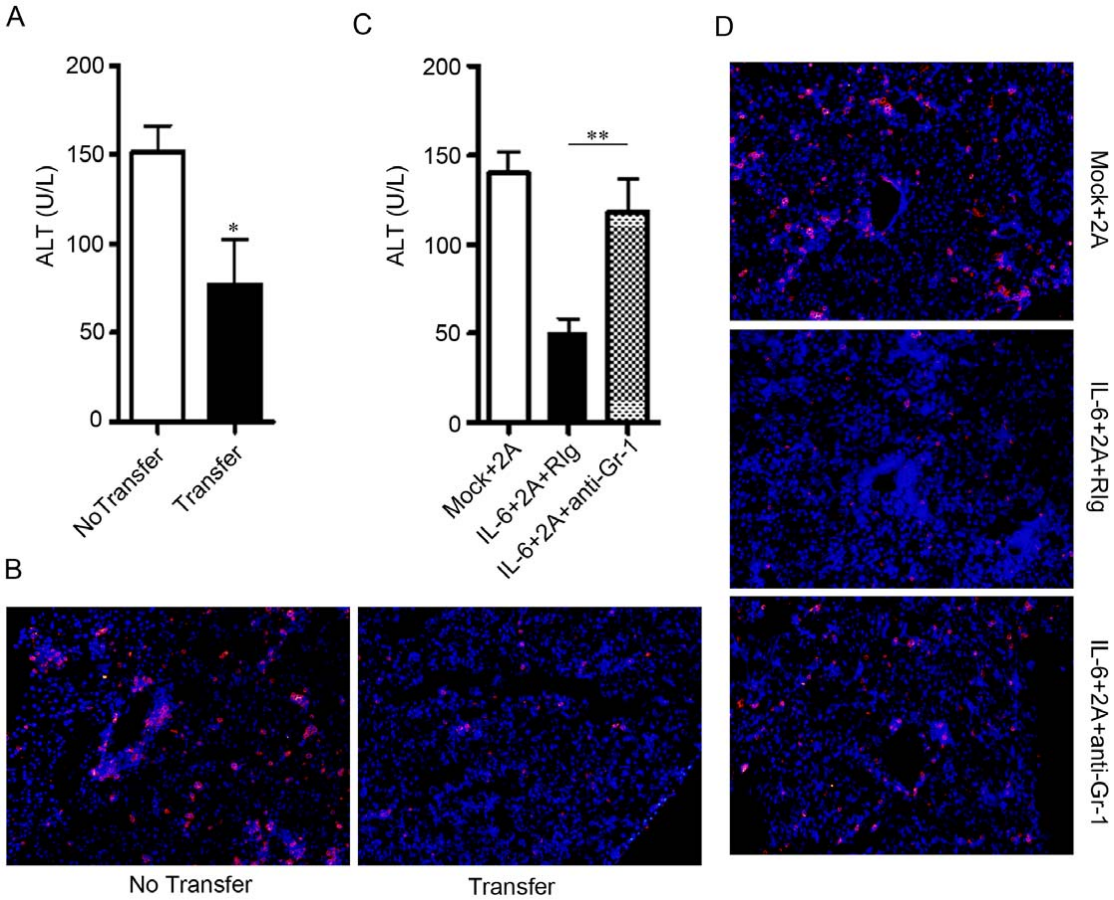
Gr-1+CD11b+ myeloid cells mediate the liver protective effect of IL-6. (A) C57BL/6 mice were i.p. injected with 100 µg 2A at day 0, and then adoptively transferred with 1×10^7^ CD11b+ intrahepatic leukocytes from IL-6 treated mice or PBS as control at day 5. ALT levels in serum were detected at day 10. CD11b+ cells purification procedure was described in *Materials and Methods*. Representative data from 2 independent experiments with at least 3 mice per group was shown. (B) Detection of CD8+ T cells (red color) in liver sections. Mice were treated as in (A). DAPI counterstaining was used to detect nuclei. (C) C57BL/6 mice were hydrodynamic injected with pcDNA-IL6 plasmid or mock plasmid at day 0. At day 2, they were i.p. injected with 2A antibody. 300 µg of anti-Gr-1 antibody or control Rat IgG were given at day 2, 4, 6, 9, and ALT levels in serum were detected at day 12. (D) Detection of CD8 T+ cells (red color) in liver sections. Mice were treated as in (C). DAPI counterstaining was used to detect nuclei. Representative data from 2 independent experiments with at least 3 mice per group is shown. *P<0.05, **P<0.01 in comparison with control groups or groups as indicated.

We investigated further whether these Gr-1+CD11b+ cells induced by IL-6 were sufficient and essential to protect 2A-induced liver injury in vivo. First, we purified CD11b+ Gr-1+ cells (>95% purity) from the liver of IL-6-treated mice and adoptively transferred into mice that were pre-treated with anti-CD137 mAb for 5 days. At day 10, we found significantly lower serum ALT level **(** **Figure 5A** **)** and less CD8+ T cells infiltration **(** **Figure 5B** **)** in mice that received Gr-1+CD11b+ cells, indicating that IL-6-induced Gr-1+CD11b+ cells can protect T cell-mediated liver injury. We also observed that the total leukocytes infiltration was also slightly decreased after transfer **(Figure S5A)**. To test whether the Gr-1+CD11b+ cells were essential for IL-6 to protect T cell-mediated liver injury, we treated mice with anti-CD137 mAb and IL-6 as above, followed by anti-Gr-1 antibody (clone RB6-8C5) injection to deplete Gr-1+ cells. The specific depletion of Gr-1+ cells were verified by FACS analysis (**Figure S5B**). Depletion of Gr-1+ cells in the 2A/IL-6 treated mice led to disappearance of leukocyte cells infiltration (**Figure S5C)**. Interestingly, the liver protective effect by IL-6 treatment was abrogated after depletion of Gr-1+ cells as shown by the significant increase of serum ALT levels **(** **Figure 5C** **)** and CD8+ T cells were significantly increased in the liver after Gr-1 depletion **(** **Figure 5D** **)**. Taken together, our data indicated that intrahepatic expression of IL-6 induced Gr-1+CD11b+ myeloid-derived suppressor cells to inhibit CD8+ T cell-mediated liver injury.

## Discussion

The role of IL-6 in T cell-mediated liver injury is not clearly defined. We report here that intrahepatic expression of IL-6 induced accumulation of Gr1+CD11b+ myeloid cells in the liver, which suppressed T cell activation. Furthermore, we showed that these myeloid cells were sufficient and critical in IL-6-mediated suppression of T cell-mediated liver injury.

IL-6 has been reported to protect acute liver injury by inhibiting hepatocyte death [22], [24]–[27]. However, its effect on CD8+ T cell-mediated liver injury has not been defined. Recently, agonist CD137 mAb treatment has been shown to lead to CD8+ T cell-mediated liver injury in both mouse and human patients. IFN-γ induction by CD8+ T cells in response to anti-CD137 mAb treatment contributed to liver injury[7]. When IL-6 was expressed in the liver via hydrodynamic delivery, it effectively inhibited the liver injury induced by the CD137 mAb. The Gr1+CD11b+ myeloid cells that were induced in IL-6-treated mice mediated this protective effect by inhibiting CD8+ T cells activation and IFN-γ induction. In the T cell suppression assay in vitro, IL6-induced Gr-1+CD11b+ cells inhibited proliferation of both CD4+ and CD8+ T cells (data not shown). However, because CD8+ T cells were preferentially increased in the liver after 2A treatment in vivo **(Figure S1B-C)**, IL-6 significantly inhibited only proliferation of CD8+ T cells but not CD4+ T cells in the liver in vivo **(** **Figure 2A** **and** **Figure 3C** **)**. Therefore we discovered a novel activity of IL-6 in protecting T cell-mediated liver injury by inducing Gr1+CD11b+ myeloid cells which then could inhibit CD8+ T cells proliferation and IFN-γ expression.

Gr1+CD11b+ myeloid cells have been reported in a number of tumor models where they have been named myeloid-derived suppressor cells (MDSCs)[31]. The immune regulation functions of MDSCs in various tumor models have gained special attention in recent years. Although most of the attention has been focused on the role of these cells in the field of cancer research, accumulating evidence has indicated that MDSCs also regulate immune responses during many other pathological conditions such infection, autoimmunity and so on[31]. However, there were only limited data on MDSCs functions in immune-mediated hepatitis. And at the same time, knowledge about factors that induced MDSCs production and their homing to peripheral tissues or lymphoid organs was incomplete. Reports indicated that several tumor-derived factors induced MDSCs expansion and most of them triggered signaling pathways in MDSCs that converge on JAK-STAT3[31]. In this paper, we gave direct evidence that IL-6 can tremendously induce the Gr1+CD11b+ myeloid cells in vivo **(** **Figure 3** **)** and these cells mediated the liver injury protection effect of IL-6. Simultaneously, a study also showed that hepatic gp130 signaling was required for mobilization and accumulation of MDSCs during polymicrobial sepsis and that hepatic acute-phase proteins serum amyloid A and Cxcl1/KC cooperatively play a role in the process [34]. Whether the MDSCs induction potency of IL-6 in our study was due to its direct effect on myeloid progenitor cells or its effect on the hepatocyte need further study. As MDSCs consist of heterogeneous, and not clearly defined, populations of myeloid lineage cells, it will be of interest to identify the specific MDSC subset in future experiments.

Elevated IL-6 expression is associated with immune-mediated liver disease progression. It likely plays a role in protecting liver injury. However, the prolonged IL-6 expression and MDSCs accumulation in the liver may have negative effect in promoting liver disease progression such as liver fibrosis and HCC development. In this respect, several studies showed that IL-6 may be a risk factor for hepatocellular carcinoma development [35]–[37]. It is also noteworthy that MDSCs have been shown to accumulate in human hepatocellular carcinoma [38]. It will be of interest to further test this hypothesis in relevant models. It will be of clinical importance to define the role of IL-6 and the “MDSCs” in T cell-mediated liver injury during viral hepatitis or autoimmune liver diseases. It is conceivable that IL-6 and the MDSCs may provide novel targets for clinical intervention in treating the immune-mediated diseases.

## Materials and Methods

### Experimental animal

Female C57BL/6 mice (aged 6–8 wk) were obtained from Weitonglihua (Beijing, China). The mice were maintained under specific pathogen-free barrier facility at the Institute of Biophysics, Chinese Academy of Sciences. All animal studies were approved by the Animal Welfare and Research Ethics Committee of the Institute of Biophysics, Chinese Academy of Sciences. Protocol No. IBP-2008104.

### Flow cytometry, in vivo BrdU labeling, and in vitro proliferation assay

The antibodies used for FACS staining including anti-mouse Gr-1-FITC, CD11b-APC, CD4-PE, CD8-FITC, NK1.1-PE, B220-FITC, CD3-APC, FoxP3-APC were purchased from eBioscience (San Diego, CA). The in vivo Gr-1+ cells depletion antibody RB6-8C5 was from BioXCell (West Lebanon, NH). The anit-CD137 mAb (clone 2A) was described previously [39] and and rat anti-KLH mAb (clone 6D11) was served as control Rat Ig. For BrdU labeling experiments, mice were given BrdU (Sigma-Aldrich, St Louis, MO) daily in the drinking water (0.8 mg/mL), and the BrdU incorporation was measured by using a BrdU Staining kit (BD Pharmingen,San Diego, CA). For in vitro proliferation assay, purified T cells were labeled with 2.5 µM CFSE (Sigma-Aldrich, St Louis, MO) and then co-cultured with various amounts of purified CD11b+ cells from the intrahepatic leukocytes (IHLs) of the IL-6 treated mice in the presence of 6 µg/ml immobilized anti-CD3 (eBioscience, San Diego, CA) for 60 hours. Two-color flow cytometric analyses were performed using a FACS Calibur flow cytometer (BD Biosciences,San Jose, CA) and analyzed using Flowjo software (Tree Star, Ashland, OR).

### Plasmid in vivo transfection

The IL-6 cDNA was cloned from LPS stimulated mouse peritoneal macrophages and inserted into pcDNA-3.1 to construct the IL-6 expression plasmid, while the mock pcDNA-3.1 was used as control plasmid in the study. The plasmids used in this study were purified by endotoxin-free plasmid DNA purification kit (Macherey-Nagel, Germany). Plasmid DNA was transfected in vivo by using a hydrodynamic- based gene transfer technique. Briefly, 10 µg/mouse DNA was diluted in 2.0 ml of PBS (∼0.1 ml/g body weight) and injected into the tail vein using a 27-gauge needle and syringe within a time period of 5 to 8 seconds. The expression of IL-6 in vivo was confirmed by detecting the IL-6 in the serum with a commercial ELISA kit from eBioscience (San Diego, CA) 24 hours after hydrodynamic injection (data not shown).

### Cell preparation

Mouse intrahepatic leukocytes (IHLs) were isolated as described previously [40]. For CD11b+ cell isolation, the CD11b+ IHLs from IL-6 treated mice were further isolated by positive selection (Miltenyi Biotec, Auburn, CA). About 95% of the purified CD11b+ cells were CD11b+ Gr-1+ cells (Data not shown). To purify T cells, splenocytes from naïve C57BL/6 mice were negative selected by exclusion of NK1.1+, Gr-1+, CD11b+, B220+ cells. The purity of CD3+ T lymphocytes after selection was over 90% as confirmed by FACS.

### Cytokine and Serum ALT Analysis

The cytokine levels in tissue homogenates of mice was analyzed using CBA inflammation kit (BD Pharmingen, San Diego, CA) on a FACS Calibur Cytometer (BD Bioscience, San Jose, CA). The serum ALT was measured by using a commercial kit (Biosino Company, Beijing, China) on Nipro Miracle ACE 919 automated biochemical analyzer (Nissho Electronics, Japan).

### Hematoxylin-eosin and immunofluorescence staining of liver sections

The livers were fixed with 4% Paraform aldehyde (PFA), embedded, sliced and further stained with hematoxylin-eosin for pathological analysis. For immunofluorescence staining, cryosections of frozen livers were performed and slides were fixed with acetone. CD8 immunofluorescence staining was performed by using rat anti-mouse CD8 antibody (Biolegend, San Diego, CA). As a second antibody, Alexa Fluor 555 goat anti-rat IgG (Invitrogen, Carlsbad, CA) was used. DAPI (BD Pharmingen,San Diego, CA) counterstaining was used to detect nuclei.

### Statistics

Statistical analyses were performed with the Graphpad prism 4 (Graphpad, San Diego,CA). Error bars represent as s.e.m. *P* value less than 0.05 was considered as significant (*), less than 0.01 or 0.001 was shown as ** or ***, respectively.

## Supporting Information

Figure S1 **2A treatment leads to intrahepatic leukocytes infiltration.** C57BL/6 mice were injected i.p. with 100 µg 2A antibody or or control antibody (RIg). At day 10, the intrahepatic leukocytes (IHLs) were isolated for FACS staining. (A) The total number of IHLs. (B) The frequency of each subset in IHLs, and (C) The number of each subset in IHLs were showed.
(TIF)

Figure S2 **Ectopic IL-6 expression in vivo leads to splenomegaly.** C57BL/6 mice were treated as in Figure 1. At day 12, the mice were sacrificed and typical spleen morphology was showed in (A). (B) Indicated spleen cell numbers in each group. Three experiments with similar results were performed. **P<0.01, ***P<0.001 in comparison with groups as indicated.
(TIF)

Figure S3 **Proliferation of CD8+ T cells is inhibited in the spleen in IL-6 treated mice.** C57BL/6 mice were treated as in Figure 2. At day12, the spleen cells were harvest and BrdU incorporation was analyzed by FACS. Percentage of BrdU+ CD8+ T cells in total CD8+ T cell in spleen and the number of BrdU+ CD8+ T cells were showed. Representative data from 2 independent experiments with at least 3 mice per group is shown. *P<0.05, **P<0.01 in comparison with control groups.
(TIF)

Figure S4 **Gr1+CD11b+ myeloid cells are dramatically increased in the spleen.** C57BL/6 mice were treated as in Figure 3. At day 12 the spleen cells were isolated for FACS analysis. (A) Representative staining of Gr-1+CD11b+ cells in the spleen. (B) The percentage of Gr-1+CD11b+ cells in spleen cells. (C) The number of CD4+ T cells, CD8+ T cells, NKT cells, NK cells, B cells, and Gr-1+CD11b+ cells in the spleen of 2A treated mice. Graphs represent the mean (SD) of 3–5 mice each group. Three independent experiments were performed with similar results. *P<0.05, **P<0.01, ***P<0.001 in comparison with control groups or groups as indicated.
(TIF)

Figure S5 **Adoptive transfer and depletion of MDSCs in vivo.** (A) C57BL/6 mice were treated as in Figure 5A. At day 10 the liver sections were collected for H&E staining. (B) Mice were treated as in Figure 5C. At day 10 the peripheral blood leukocytes of the mice were collected and stained for Gr-1+ CD11b+ cells. (C) Mice were treated as in Figure 5C. At day12, the livers were fixed and stained with H&E.
(TIF)
