# Supplementary figures and images for: Interleukin-6 Induces Gr-1+CD11b+ Myeloid Cells to Suppress CD8+ T Cell-Mediated Liver Injury in Mice
Source: PLoS One. 2011 Mar 4;6(3):e17631. doi: 10.1371/journal.pone.0017631 (PMC3048877; doi:10.1371/journal.pone.0017631)

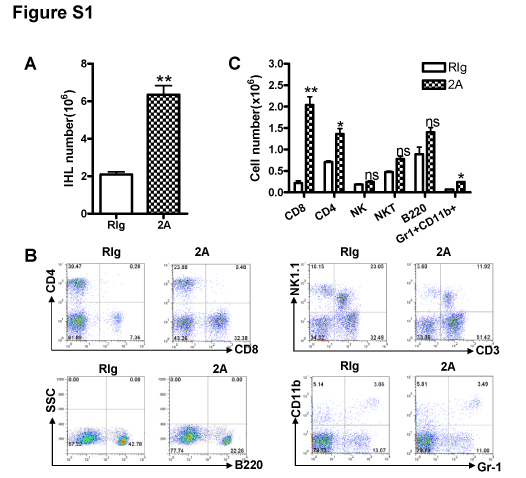

Supplement: Figure S1 — 2A treatment leads to intrahepatic leukocytes infiltration. C57BL/6 mice were injected i.p. with 100 µg 2A antibody or or control antibody (RIg). At day 10, the intrahepatic leukocytes (IHLs) were isolated for FACS staining. (A) The total number of IHLs. (B) The frequency of each subset in IHLs, and (C) The number of each subset in IHLs were showed. (TIF) [file pone.0017631.s001.tif]

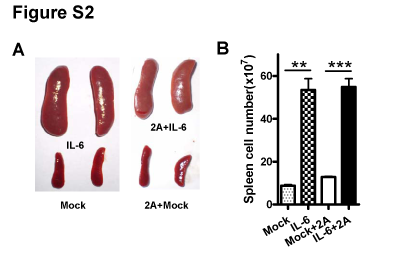

Supplement: Figure S2 — Ectopic IL-6 expression in vivo leads to splenomegaly. C57BL/6 mice were treated as in Figure 1. At day 12, the mice were sacrificed and typical spleen morphology was showed in (A). (B) Indicated spleen cell numbers in each group. Three experiments with similar results were performed. **P<0.01, ***P<0.001 in comparison with groups as indicated. (TIF) [file pone.0017631.s002.tif]

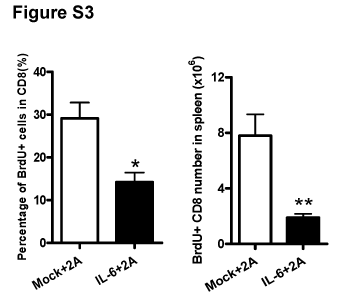

Supplement: Figure S3 — Proliferation of CD8+ T cells is inhibited in the spleen in IL-6 treated mice. C57BL/6 mice were treated as in Figure 2. At day12, the spleen cells were harvest and BrdU incorporation was analyzed by FACS. Percentage of BrdU+ CD8+ T cells in total CD8+ T cell in spleen and the number of BrdU+ CD8+ T cells were showed. Representative data from 2 independent experiments with at least 3 mice per group is shown. *P<0.05, **P<0.01 in comparison with control groups. (TIF) [file pone.0017631.s003.tif]

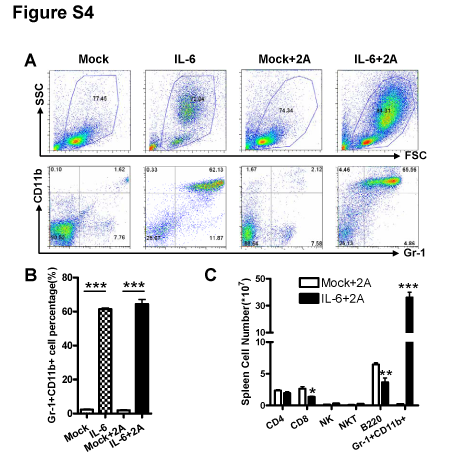

Supplement: Figure S4 — Gr1+CD11b+ myeloid cells are dramatically increased in the spleen. C57BL/6 mice were treated as in Figure 3. At day 12 the spleen cells were isolated for FACS analysis. (A) Representative staining of Gr-1+CD11b+ cells in the spleen. (B) The percentage of Gr-1+CD11b+ cells in spleen cells. (C) The number of CD4+ T cells, CD8+ T cells, NKT cells, NK cells, B cells, and Gr-1+CD11b+ cells in the spleen of 2A treated mice. Graphs represent the mean (SD) of 3–5 mice each group. Three independent experiments were performed with similar results. *P<0.05, **P<0.01, ***P<0.001 in comparison with control groups or groups as indicated. (TIF) [file pone.0017631.s004.tif]

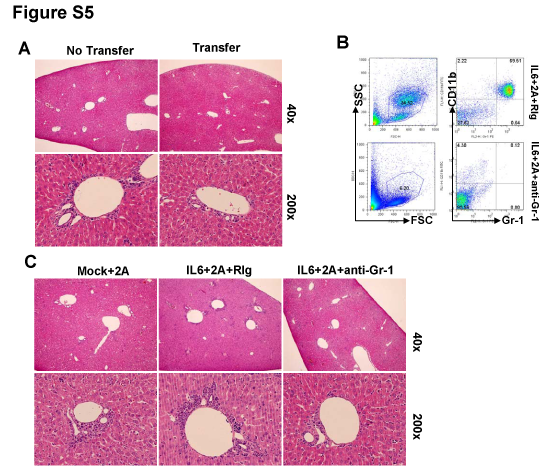

Supplement: Figure S5 — Adoptive transfer and depletion of MDSCs in vivo. (A) C57BL/6 mice were treated as in Figure 5A. At day 10 the liver sections were collected for H&E staining. (B) Mice were treated as in Figure 5C. At day 10 the peripheral blood leukocytes of the mice were collected and stained for Gr-1+ CD11b+ cells. (C) Mice were treated as in Figure 5C. At day12, the livers were fixed and stained with H&E. (TIF) [file pone.0017631.s005.tif]
